# Iranian nurses' knowledge, attitude and behaviour on skin care, prevention and management of pressure injury: A descriptive cross‐sectional study

**DOI:** 10.1002/nop2.365

**Published:** 2019-09-12

**Authors:** Mojgan Lotfi, Ahmad Mirza Aghazadeh, Hossein Asgarpour, Afsaneh Nobakht

**Affiliations:** ^1^ Department of Medical Surgical Nursing, Faculty of Nursing and Midwifery, Sina Hospital Tabriz University of Medical Sciences Tabriz Iran; ^2^ Department of Basic sciences, Paramedical Faculty Tabriz University of Medical Sciences Tabriz Iran; ^3^ Department of Internal Surgery Faculty Member of Çanakkale onsekiz mart Çanakkale Turkey; ^4^ Faculty of Nursing and Midwifery, Sina Hospital Tabriz University of Medical Sciences Tabriz Iran

**Keywords:** attitude, knowledge, nurses, nursing, pressure injury

## Abstract

**Aim:**

Pressure injuries as an indicator measuring the quality of nursing care and patient safety is a major health care problem worldwide. The aim of this study was to assess the knowledge, attitude and behaviour of nurses in preventing pressure injuries.

**Design:**

Descriptive cross‐sectional study.

**Methods:**

This descriptive cross‐sectional study enrolled 214 registered nurses in Iran. Patient satisfaction was assessed using pieker pressure ulcer knowledge test, attitude towards pressure ulcer tool and behaviour of pressure ulcer questionnaire. Data analysed by SPSSv.24 applying descriptive and inferential statistics.

**Results:**

The mean scores of knowledge, attitude and behaviour of nurses on the prevention of pressure injury were 27.24 (*SD* 5.23), 38.55 (*SD* 6.43) and 51.24 (*SD* 7.54), respectively. There was a correlation between knowledge, attitude and behaviour with the history of pressure injury training. Also, there was a significant relationship between knowledge with educational level and attitude with work experience. Knowledge, attitude and behaviour of nurses were in moderate level. Necessary measures to overcome problems such as the availability of pressure reducing equipments, motivating the nurses, eliminating the shortage of nurses and empowering nurses by holding practical workshops are important in providing patients safety.

## 1 INTRODUCTION

Pressure injury is a common, painful, costly and possibly preventable complication in health care centres (Gunningberg et al., 2015). International Organization of Pressure Ulcer Advisory Panel (NPUAP) defined it as the local injury of the skin or the underlying tissues that occurs around a bony prominence as a result of the pressure or composition of the pressure and the slipping forces of friction (Edsberg et al., 2016). Pressure injury is often seen in patients with physical disabilities, people with impaired mobility and admission to special sectors and is recognized as an international problem (Beeckman, Defloor, Schoonhoven, & Vanderwee, 2011) affecting more than 1.3 million adults worldwide every year (Ghanee, 2012). The incidence of pressure injury varies from country to country, with an estimated 14%–17% in the United States, 18.1% in the European countries and 19% in Iran (El Enein & Zaghloul, 2011; Karimian, Sarokhani, Sarokhani, Sayehmiri, & Mortazavi Tabatabai, 2016).

Pressure injuries will have adverse effects, such as pain, infection, increased hospitalization time and treatment costs, reduced quality of life, increased mortality and legal consequences (Kula & GaPUD, 2018). In British health services, the annual cost of treatment for pressure injury is between 1.4–2.1 billion pounds (Papanikolaou, Lyne, & Anthony, 2007) and in the United States about 2 billion and 200 million dollars (Brindle & Wegelin, 2012). Despite the advances in timely diagnosis and wound healing and despite the international wound guides and the quality of health care provision, the patient's pressure injury remains high, and it is a worldwide problem (Jackson et al., 2016). The American Nurses Association cites pressure injury as an indicator of the quality of safety assessment and nursing service quality index in the hospital environment (Worsley, Clarkson, Bader, & Schoonhoven, 2017). Meanwhile, although a multidisciplinary team approach plays a key role in the prevention of pressure injuries, nurses are at the forefront of the prevention and treatment of pressure injuries in the health system (Köse & Öztunç, 2016).

They are directly involved in key aspects of preventing pressure injury such as risk assessment (Hekmatpou, Mehrabi, Rahzani, & Aminiyan, 2018). Because of the acute impact of nurses on this, more pressure injuries can be prevented (Etafa, Argaw, Gemechu, & Melese, 2018). Nurses are not fully aware of daily wound care protocols and may not have sufficient knowledge about evidence‐based practices. Occasionally, nurses’ behaviours are not knowledge‐based, but based on experience or habit (Dalvand, Ebadi, & Gheshlagh, 2018). They need to have good knowledge, skills and behavioural beliefs in this field to provide qualified and effective care. The nurse's knowledge of pressure injury is essential for risk assessment, risk management and implementation of preventive approaches (Parvan, Hosseini, & Bagherian, 2018). According to studies conducted in Belgium, Sweden and Turkey, the level of nurses' knowledge of pressure injury prevention has been reported inadequate (Dalvand et al., 2018; Etafa et al., 2018; Papanikolaou et al., 2007).

In a study conducted in Iran by Farzi, Yousefii, Moladoost, & Moieni (2016), the results indicated a low level of knowledge and attitude of nurses and thus their inadequate knowledge of their performance in preventing injury pressure. Inadequate knowledge and non‐compliance of nurses with standards and guidelines that govern the management of injury are a common problem in preventing and managing them (Simonetti, Comparcini, Flacco, Di Giovanni, & Cicolini, 2015). To eliminate barriers to prevention, authorities should have more information about the knowledge, attitude and behaviour of nurses in relation to the standards of prevention of pressure injury, to communicate executive and operational guidelines and make nurses responsible for providing care (Dilie & Mengistu, 2015).

Considering the importance of prevention and management of pressure injury and considering that the information necessary for decision‐making for nurses' empowerment programs and prevention of pressure injury were not found in Tabriz University of Medical Sciences, this study aimed at evaluating the knowledge, attitude and behaviour of nurses about skin care, prevention and management of pressure injury in educational centre of Tabriz.

## 2 METHODS

### 2.1 Design

It was a descriptive cross‐sectional study carried out in the form of a research proposal approved by Tabriz University of Medical Sciences from November 2018 to February 2019.

### 2.2 Setting and participants

After receiving permission from university officials and management, introducing the goals of the study to the authorities of the relevant departments and obtaining the ethical code (1397.53IR.TBZMED.REC.), nurses working in educational centres affiliated to Tabriz University of Medical Sciences was invited to participate in the study. Confidentiality of information was assured to them. The population consisted of all nurses in the internal, surgical and specialist departments of educational hospitals (Sina, Shohada, Imam Reza, Madani) of Tabriz University of Medical Sciences (1,134 people), among whom 214 were selected as sample size. Simple random sampling was used for this purpose. Sampling was done according to the available list of nurses working in hospitals; each of nurses was selected randomly for inclusion in the study.

$$n=\frac{Npqz^{2}}{\left(N-1\right)E^{2}+pqz^{2}}\;p=0.78;q=0.22;N=1,134$$

$$n=\frac{1,134\left(0.78\right)\left(0.22\right){\left(1.96\right)}^{2}}{\left(1,133\right){\left(0.05\right)}^{2}+\left(0.78\right)\left(0.22\right){\left(1.96\right)}^{2}}≅214$$

### 2.3 Data collection and procedures

Criteria for entering the study: nurses working in the internal, surgical and special sectors, nurses who are willing to participate in the research and nurses with a working experience of at least 6 months and exit criteria including the following: nurses' unwillingness to continue cooperation and leaving the research.

To collect information, a four‐part questionnaire was used including the following:

1. Demographic information of nurses including age, sex, level of education, work experience and history of learning about pressure injury.
2. Pieker Pressure Ulcer Knowledge Test (PPURT), which included 41 items asking about nurses' knowledge about the three areas of how a pressure injury starts, the characteristics of pressure injury and the prevention of pressure injury (yes/no question). According to this tool, knowledge of nurses is considered sufficient enough when they answer 90% or more of the questions correctly. After scoring, the results were calculated and expressed on a scale of 100. This tool has three levels of desirable (70% and above), relatively desirable (50%–69%) and undesirable (less than 50%) (Pieper & Mott, 1995).
3. Attitude towards Pressure Ulcer Tool (APUP) which included 10 questions. The answer to the field of attitude includes a Likert scale from completely agree (5), agree (4), I have no opinion (3), disagree (2) and completely disagree (1). The results were calculated as 100 and were categorized in three levels of negative attitude (less than 71%), moderate attitude (71%–84%) and positive attitude (above 84%) (Moore & Price, 2004).
4. Behaviour of pressure ulcer questionnaire that included 22 items. Responses included always (3), sometimes (2) and never (1) options. Results were calculated on a scale of 100 at very low levels (less than 60%), low (60%–69%), moderate (70%–79%), high (89%–80%) and very high (90%–99%).To investigate the validity of the questionnaire, content validity has been used. For this purpose, the tool was given to eight faculty members who were professors of Tabriz University of Medical Sciences. The content of the questions was evaluated and judged and after the collection of comments, changes were considered.

### 2.4 Statistical analysis

To confirm the reliability of the questionnaires, test re‐test criteria were used. For this purpose, the questionnaires were given to 10 nurses working in the educational centres within two weeks. Correlation coefficient between two test scores was 0.85. Data were analysed by descriptive statistics (frequency and percent, mean and standard deviation) and inferential statistics (*t* test and ANOVA, Mann–Whitney, Kruskal–Wallis and Chi‐square tests) using SPSS 24 software at a significant level *α* = .5.

## 3 RESULTS

From 240 distributed questionnaires, 214 questionnaires were completed and analysed. 88.3% of participants were female with a mean age of 31.95 (*SD* 6.56) years and a working experience 7.35 (*SD* 6.20) years. Most of the respondents had an undergraduate degree (89.7%); only 36% of the subjects had undergone training for pressure injury. Table 1 shows the distribution of nurses participating in the study.

**Table 1:** Frequency distribution of nurses in teaching hospitals affiliated to Tabriz University of Medical Sciences

Variables	*N* (%)
Sex	
Male	25 (11.7)
Female	189 (88.5)
Age	
<31	100 (46.7)
31–41	96 (44.9)
>41	18 (8.4)
Educational status	
BSc.	192 (89.7)
MSc.	22 (10.5)
Working experience (years)	
<5	108 (50.5)
5–10	44 (20.6)
10–14	28 (13.1)
>14	34 (15.9)
Clinic (now working)	
Internal clinics	60 (28.0)
Surgical clinics	52 (24.5)
Intensive care units	102 (47.7)
The case of having training about pressure ulcer	
Yes	77 (36.0)
No	137 (64.0)

Shapiro‐Wilk test showed that knowledge (*p* = .0058) and attitude (*p* = .0051) variables had abnormal distribution, and the other variables follow the normal distribution. The mean scores of knowledge and attitude and behaviours were 27.23 (*SD* 5.23), 38.55 (*SD* 6.43) and 51.28 (*SD* 7.54), respectively. The results showed that the mean score of knowledge, attitude and behaviour of nurses regarding pressure injury was relatively moderate and favourable. Regarding the abnormal distribution of knowledge and attitude score and normal distribution of behaviour score, Mann–Whitney *U* and Kruskal–Wallis tests, One‐way ANOVA and Independent *t* tests were used. According to Kruskal–Wallis test, nurses' knowledge scores in different levels of working experience were not significantly different (*p* = .207).

The Mann–Whitney test showed that nurses' knowledge score had a significant difference with educational level (*p* = .041) and training history (*p* = .0001). Kruskal–Wallis test showed that nurses' attitude score had significant differences in different levels of working experience (*p* = .019). According to the Mann–Whitney test, the mean score of attitudes in people with working experience of over 14 years was higher than those with less than 5 years and 10–14 years. According to the Mann–Whitney test, nurses' attitude score did not show significant differences with educational level (*p* = .359). The Mann–Whitney test showed a significant difference between the attitude of nurses with training history (*p* = .0001).

According to ANOVA, there was no significant difference between the behaviour score of nurses in different working experience groups (*p* = .081, *F* = 2.275). Independent *t* test showed no significant difference between behaviour score and educational level (*p* = .341). According to independent *t* test, there was a significant difference between the behaviour scores of nurses with training history (*p* = .0001) (Table 2).

**Table 2:** Comparison of mean scores of knowledge, attitude and behaviour of nurses in terms of demographic characteristics

Variables	*N*	Knowledge			Attitude			Behaviour		
		Mean	*SD*	*p*	Mean	*SD*	*p*	Mean	*SD*	*p*
Working experience										
<5	108	26.59	5.24	.207	37.89	5.86	.019	50.01	7.88	.085
5–10	44	28.29	4.97		38.63	6.96		53.11	8.26	
10–14	28	27.82	4.23		37.53	6.22		52.50	6.29	
>14	34	27.47	6.15		41.32	7.14		51.91	5.74	
Educational status										
BSc.	192	27.02	5.18	.041	38.43	6.26	.359	51.10	7.76	.341
MSc.	22	29.13	5.40		39.13	7.92		52.81	5.11	
The case of having training about pressure ulcer										
Yes	77	30.66	3.21	<.0001	42.88	7.05	<.0001	53.85	5.87	<.0001
No	137	25.32	5.18		36.12	4.53		49.83	8.00	

Chi‐square test was used to examine the relationship between knowledge, attitude and behaviour variables. The findings showed a significant correlation between knowledge, attitude and behaviour, but no significant relationship was found between attitude and behaviour (Table 3).

**Table 3:** Relationship between knowledge, attitude and behaviour of nurses regarding pressure injury

Variables	Attitude	Behaviour
Knowledge	*χ* ^2^ = 37.814 *df* = 4; *p*=<.0001	*χ* ^2^ = 24.135 *df* = 8; *p* = .002
Attitude		*χ* ^2^ = 11.986 *df* = 8; *p* = .152

## 4 DISCUSSION

Nurses are key members of the health team and the largest part of the professional force providing services. Therefore, they must be aware of potential risk factors and preventive measures for pressure injury and their performance should be based on the best scientific evidence (Claudia, Diane, Daphney, & Danièle, 2010).

The results of this study showed that knowledge of nurses on pressure injury was relatively desirable. However, in a descriptive study conducted by Gunningberg et al. (2015), examining the knowledge of nurses and nursing students in preventing pressure injury in Sweden, the results showed an insufficient knowledge. Other studies on the knowledge of nurses about pressure injury showed that their knowledge was inadequate (Al Kharabsheh, Alrimawi, Al Assaf, & Saleh, 2014; Chianca, Rezende, Borges, Nogueira, & Caliri, 2010; Tirgari, Mirshekari, & Forouzi, 2018). The difference in the results of this study can be related to the differences in the population.

In examining this area, most nurses answered correctly this item: “People with pressure ulcer should be on the pressure reducing surfaces”. Moreover, the items of applying pressure injury rehabilitation programs, risk factors and ways to prevent pressure injury had the most correct answers among nurses.

Part of lack of knowledge of nurses can be due to a lot of work and the inability to participate in training programs on pressure injury (Soozani et al., 2012). According to the findings of this study, 64% of nurses did not participate in educational pressure injury training programs. The findings of this study showed that nurses' attitude towards pressure injury was in the moderate level. Based on the findings of this study, nurses believed that they should not be concerned about the prevention of pressure injury in their work. Low knowledge about pressure injury seems to justify the inappropriate attitude of nurses in preventing it. The level of attitude of nurses was relatively favourable in the study by Farzi et al. (2016). In a cross‐sectional study by Bayan Kaddoura et al., the nurses' attitude towards pressure injury has been addressed and the moderate attitude level has been reported, which is in line with this study (Kaddourah, Abu‐Shaheen, & Al‐Tannir, 2016).

Usher et al. (2018) showed that students had a positive attitude towards prevention of pressure injury which is not consistent with this study. The difference can be related to the difference in the studied population. The level of nurses' behaviour was moderate in the present study, which is consistent with the results of the Källman & Suserud study who examined the knowledge, attitude and behaviour of nurses on prevention of pressure injury (Källman & Suserud, 2009). According to the results, the behaviour of nurses had a significant relationship with the history of training. It can be concluded that the relatively acceptable performance of nurses can be due to their limited information on the important parameters of creating a pressure injury and the lack of use of pressure reducing equipment and risk assessment tools (Soozani et al., 2012).

According to the results, the nurses gave the lowest correct answers to “To prevent pressure ulcer, I do not use donut‐shaped pillows (ring pillows) in the bone prominence area”. The findings showed that nurses were less interested in applying pressure relief levels for patients. This may be due to their lack of awareness of the use of them.

On the other hand, the results showed that there was a significant relationship between knowledge and attitude and behaviour of nurses in prevention of pressure injury. However, there was no significant relationship between nurses’ attitude and behaviour.

It is believed that all three areas of knowledge, attitude and behaviour should be strengthened to create the desired effects. Just knowledge does not lead to good behaviours, but attitudes must be changed, and the structure of people's beliefs must be deepened and scientifically established to function properly (Saifollahi, Bolourchifard, Borhani, Ilkhani, & Jumbarsang, 2016).

In fact, it is necessary to plan measures to increase the knowledge and awareness of nurses and other therapeutic personnel in this regard, so that they have a positive attitude and this attitude can lead to a favourable and appropriate behaviour in prevention and management of pressure injury and ultimately improve patient safety (Whyte, Ward, & Eccles, 2009).

Moore and Price emphasized that in addition to the positive attitude of nurses regarding the prevention of pressure injury, there is a difference between theory and behaviour due to insufficient human resources and lack of time (Moore & Price, 2004). Lack of proper motivation for nurses with sufficient knowledge due to job dissatisfaction and delays in nurses' demands are among these. Therefore, increased motivation by the authorities for nurses who have good knowledge in this field (care and prevention of pressure injuries) can help implementation of standards for prevention of pressure injury in the departments (Mwebaza, Katende, Groves, & Nankumbi, 2014).

## 5 STUDY LIMITATIONS

During the data collection, lack of nursing staff, lack of time, lack of motivation and lack of interest in management and prevention of pressure injury and dissatisfaction with management and payment systems were the ones that nurses expressed as the reasons for their unwillingness to participate in the study. The researcher tried to convince them to participate in the study by discussing and explaining the importance of the results for the education and training of nurses.

## 6 CONCLUSION

In this study, although knowledge, attitude and behaviour of nurses in preventing pressure injury were at a relatively good and moderate level, they are still far from the desirable level. Considering the importance of patient safety and its improvement, it is necessary to pay more attention to the education of people in health services in the field of prevention standards for pressure injury and the necessary measures to overcome problems such as availability of stress relief equipment in all centres, nursing motivation, eliminating the shortage of capable nurses and holding workshops should be considered.

Due to the fact that pressure injury is one of the most dangerous threats to the patient's safety, holding continuous training courses to raise awareness and reinforce positive attitudes in them and correct some of the wrong actions as a result of false beliefs for nurses and other medical personnel in hospitals seems essential. Creating motivation in nurses with different management methods and coordination between physicians and nursing colleagues in the therapeutic programs on the one hand and in lining the training on the prevention and care of pressure injuries on the agenda, on the other hand, are very important.

## CONFLICTS OF INTEREST

None.

## COMPLIANCE WITH ETHICAL STANDARDS

The ethics committee of Tabriz University of medical sciences authorized the permission to conduct this study (Ethical no is IR.TBZMED.REC.1397/53). All of the authors have full control of all primary data, and they agree to allow the journal to review their data if requested.
